# Genetic architecture and candidate genes detected for chicken internal organ weight with a 600 K single nucleotide polymorphism array

**DOI:** 10.5713/ajas.18.0274

**Published:** 2018-07-26

**Authors:** Taocun Dou, Manman Shen, Meng Ma, Liang Qu, Yongfeng Li, Yuping Hu, Jian Lu, Jun Guo, Xingguo Wang, Kehua Wang

**Affiliations:** 1 Jiangsu Institute of Poultry Science, Chinese Academy of Agricultural Sciences, Yangzhou, Jiangsu 225216, China; 2 College of Animal Science and Technology, Yangzhou University, Yangzhou, Jiangsu 225009, China; 3 College of Animal Science and Technology, Nanjing Agriculture University, Nanjing, Jiangsu 210095, China

**Keywords:** Internal Organ, Genome-wide Association Study, Quantitative Trait, Chicken, Fitness Trait

## Abstract

**Objective:**

Internal organs indirectly affect economic performance and well-being of animals. Study of internal organs during later layer period will allow full utilization of layer hens. Hence, we conducted a genome-wide association study (GWAS) to identify potential quantitative trait loci or genes that potentially contribute to internal organ weight.

**Methods:**

A total of 1,512 chickens originating from White Leghorn and Dongxiang Blue-Shelled chickens were genotyped using high-density Affymetrix 600 K single nucleotide polymorphism (SNP) array. We conducted a GWAS, linkage disequilibrium analysis, and heritability estimated based on SNP information by using GEMMA, Haploview and GCTA software.

**Results:**

Our results displayed that internal organ weights show moderate to high (0.283 to 0.640) heritability. Variance partitioned across chromosomes and chromosome lengths had a linear relationship for liver weight and gizzard weight (R^2^ = 0.493, 0.753). A total of 23 highly significant SNPs that associated with all internal organ weights were mainly located on *Gallus gallus* autosome (GGA) 1 and GGA4. Six SNPs on GGA2 affected heart weight. After the final analysis, five top SNPs were in or near genes 5-Hydroxytryptamine receptor 2A, general transcription factor IIF polypeptide 2, WD repeat and FYVE domain containing 2, non-SMC condensin I complex subunit G, and sonic hedgehog, which were considered as candidate genes having a pervasive role in internal organ weights.

**Conclusion:**

Our findings provide an understanding of the underlying genetic architecture of internal organs and are beneficial in the selection of chickens.

## INTRODUCTION

The internal organs are involved in many biological occurrences, including oxygen transportation, lipid metabolism, and digestion. Each organ has its own developmental process and gene expression profile [1]. Liver development affects abdominal fat, yolk deposit [2] which are all associated with economic performance, and provides a model of non-alcoholic steatohepatitis [3]. Heart is an organ for carrying oxygen [4], affect well-being of the chicken [5]. Physical and chemical digestion takes place in the proventriculus and gizzard, which regulate feed intake and energy balance [6]. Efficient utilization of feed is essential to reduce feed cost and improve economic benefit. Moreover, internal organ like gizzard and heart provide proximate composition and amino acid same as chicken meat [7]. In order to support an economical viable meat system, it is essential to efficient utilize these byproducts. Hence, improve these internal organs will not only improve well-being of the organism but also take full advantage of byproduct from chicken.

With the development of deep sequencing and statistical methods, it is possible to detect the quantitative trait loci (QTLs) of complex traits. In recent years, great advances have been gained in the selection of economic traits in chickens with molecular biology techniques. Nones et al [8] identified novel QTLs associated with gizzard, liver, heart, lung, and thigh located on *Gallus gallus* autosome (GGA) 1. Rosário et al [9] found novel QTLs on GGA1, GGA3, and GGA4 related with carcass traits. Boschiero et al [10] identified microsatellite markers associated with performance and carcass traits on GGA1 and GGA13. Most studies on chicken internal organ weight have focused on the early stages of development, which has hindered the progress of similar research in the layer hen, especially for spent layer hens in China.

In this study, we conducted a genome-wide association study (GWAS) on an F2 resource population generated from Dongxiang Blue-Shelled (DX) and White Leghorn (WL) chickens with a 600 K Affymetrix chip. Furthermore, a linkage disequilibrium (LD) analysis, variance estimated percentage, and gene annotation were performed by biotechnology. This research will provide the genetic architecture that underlies the weight of internal organs in layer chickens.

## MATERIALS AND METHODS

### Ethical statement

Animal procedures and sample collections were carried out following the guidelines established by the Ministry of Agriculture of China. The Institution of Animal Care and Use Committee in the Poultry Institute, Chinese Academy of Agricultural Science, Yangzhou, China approved the procedures.

### Phenotype collection and analyses

We constructed an F_2_ resource population by reciprocal crosses between WL and DX chickens. The WL was from a commercial line and DX from a Chinese indigenous strain, therefore the two lines represent two very different genetic pools, including morphological, physiological, and production traits, such as feed efficiency [11], follicle number [12], and the yolk proportion [13]. The F2 resource population of 1512 birds from 49 half-sib and 590 full-sib families were produced from a WL/DX (25 ♂:407 ♀) and DX/WL (24 ♂:235 ♀) cross in the F_1_ generation. The F_1_ population was generated from WL (6 ♂)×DX (133 ♀) and DX (6 ♂)×WL (80 ♀) by initial reciprocal crosses, producing 1,029 and 552 chicks, respectively. The experimental animals were kept indoors under standardized conditions at the research base in the Jiangsu Institute of Poultry Science, Yangzhou, China. Birds had *ad libitum* access to feed and water that met all NRC requirements and were housed in single-hen cages under a 16 L:8 D lighting regime. The F_2_ birds were slaughtered at 72 weeks; immediately after the slaughter we measured the weights of heart (HW), liver (LW), proventriculus (PW), and gizzard (GW). DNA was collected by standard venipuncture. Data including a descriptive phenotype and normality test statistics were summarized using R software.

### Genotyping and quality control

The standard phenol/chloroform method was used to extract genomic DNA, which was genotyped against a 600 K Affymetrix Axiom Chicken Genotyping Array (Affymetrix, Inc. Santa Clara, CA, USA). Affymetrix Power Tools v1.16.0 (APT) (http://affymetrix.com/) software was used to analyze genotype calling and quality control (QC). A total of 1,512 hens and 532,299 single nucleotide polymorphisms (SNPs) remained valid after the application of APT for QC. An R script was used to calculate the SNP QC metrics and filter out individual SNPs falling below given thresholds. The effectiveness of the detecting quality was tested by PLINK v1.90 [14]. We imputed for some sporadic missing genotypes using the BEAGLE v4.0 package [15]. Briefly, samples with a dish QC of more than 0.82, call rate less than 97%, minor allele frequency (MAF) higher than 5%, Hardy–Weinberg equilibrium test P of more than 1×E10^−6^ were kept for further analysis. After QC, 1,512 individuals and 435,867 SNPs were used in further GWA analysis.

### Genome-wide association analysis

A principal component analysis was used to eliminate spurious associations resulting from the presence of cryptic relatedness or hidden population stratification. Then, the full SNP set to 41,130 independent SNPs were pruned via the –indep-pairwise 25 5 0.2 command (PLINK), and we included five principal components (PCs) as covariates in the mixed model. After simpleM was used to determine the threshold by correcting the number of multiple tests, the genome-wide significant and suggestive p-values were 8.43×10^−7^ (0.05/59,308) and 1.69×10^−5^ (1.00/59,308), respectively.

The GEMMA v0.94 package [16], with the exact mixed model approach, was implemented with the valid individuals and SNPs for univariate analysis. The independent SNPs were used to compute the centered relatedness matrix, and the significance p-value level between SNPs and phenotypes was calculated from the derived Wald test. The univariate linear mixed model was denoted as:

y=Wα+xβ+u+ɛ

Where y represents an n-vector of phenotypic values for n individuals; W is a matrix of covariates (fixed effects with a column of 1s and top five PCs), α is a vector of the corresponding coefficients including the intercept; x is an n-vector of the genotypes of the SNP marker, β is the effect size of the marker; μ is an n-vector of random effects; and ɛ is an n-vector of errors.

The Manhattan plots and quantile–quantile (QQ) plots were drawn by “gap” packages in R software. The genomic inflation factor was calculated by using the GenABEL package in the R project.

### Linkage disequilibrium analysis and gene annotation

The LD were implemented by Haploview v4.2 to test the association of significant SNPs with each other. When the distance between two consecutive genome-wide significant SNPs was greater than 10 Mb, they were considered as two separate QTL. The LD analyses would find a haplotype blocks because of these loci located in a strong LD region. But, a genuine causal locus could not be distinguished by present GWAS method. Therefore, a functional gene annotation on these significant SNPs were essential performed to characterize potential candidate genes. In our study, we searched candidate genes based on *Galgal5* assembly, using BioMart system supported by Ensemble (http://asia.ensembl.org/Gallus_gallus/Info/Index). The genes nearest or harboring significant SNPs of internal organ weight were chosen as candidate locations.

### SNP effects and chromosome heritability

The contributions to phenotypic variance (CPV) explained by the significant association SNPs was calculated with GCTA software [17] using univariate restricted maximum likelihood. The heritability explained by the eligible SNPs (*h^2^_snp_*) for GWAS or the variance contributed by each chromosome were also estimated with GCTA.

## RESULTS

### Phenotypic statistics and genetic parameters

Descriptive data for each organ are displayed in Table 1. After rank-based inverse normal transformation, all phenotypic values conformed to a normal distribution. The range of all internal organ is large, and with the coefficient of variance more than 19%. Table 2 displayed the genetic analysis, which showed that the heritability of internal organs was moderate to high, and the GW had the highest heritability (0.640). Genetic correlation between each organ weight was higher than 0.5, the highest genetic correlation was PW and GW (0.711).

### Genome-wide association study results

**Figure 1:**
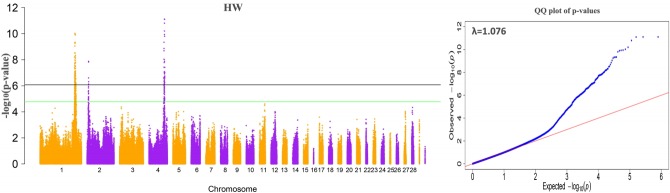
Manhattan plot and QQ-plot of genome-wide association analysis for heart weight. The left plot is the Manhattan plot, which shows the −log10 (observed p values) for association of single nucleotide polymorphisms (y-axis) plotted against their chromosomal positions on each chromosome (x-axis). The right plot is the QQ test for population structure, the x-axis indicates the expected −log10-transformed p values, and the y-axis shows the observed −log10-transformed p values.

**Figure 2:**
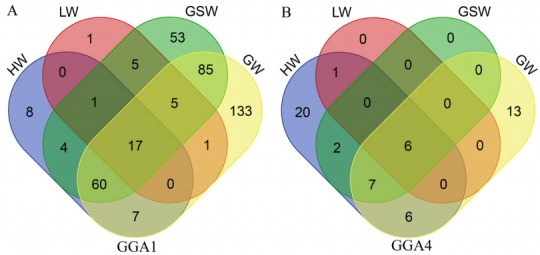
Venn diagram of significant single nucleotide polymorphisms on GGA1 (A) and GGA4 (B) associated with four internal organ weights by univariate association test. Heart weight, liver weight, proventriculus weight, and gizzard weight are abbreviated as HW, LW, PW, and GW, respectively. GGA, *Gallus gallus* autosome.

The results of the GWAS were as follows. There are 380 significant SNPs on GGA1, 49 SNPs on GGA4 were related to internal organ weights, and six SNPs on GGA2 were related to HW (Table 2, Figure 1, Supplementary Figure S1, S2, S3). The regions ranged from 165.63 to 173.78 Mb on GGA1, and from 71.30 to 77.19 Mb on GGA4. After Venn diagram analysis, 17 and 6 significant SNPs located on GGA1 and GGA4, respectively, were found to affect all internal organ weights (Figure 2).

### Estimation of contributions to phenotypic variance and plausible candidate genes

By using gene annotation analysis, it was obtained that the 17 significant SNPs on GGA1 were harbored in nine genes, general transcription factor IIF polypeptide 2 (*GTF2F2*), potassium channel tetramerization domain containing 4, spermatid associated, succinate-coA ligase ADP-forming β subunit, 5-hydroxytryptamine receptor 2A (*HTR2A*), WD repeat and FYVE domain containing 2 (*WDFY2*), asparagine-linked glycosylation 11, NIMA related kinase 3, and cytoskeleton associated protein 2. The SNP on GGA1 possessing a MAF of more than 0.4 and the values of beta on GGA1 were all negative (Table 4). We then analyzed the allelic contribution to phenotypic variation (Table 4), the results show that the smaller the p value, the larger the CPV value. All significant SNPs on GGA1 explained over 5% of CPV for PW and GW, while the CPV for HW and LW was from 2.781% to 4.723%. The CPV of SNPs *rs312726815*, *rs315120631*, *rs13972990* were highest for HW, LW, and PW/GW. The *rs312726815* SNP located in the upstream (16,831 bp) of *HTR2A*, *rs315120631* located on the intron of *GTF2F2*, *rs13972990* was downstream (95,255 bp) of *WDFY2*.

Table 5 displayed that the SNPs on GGA4 possess a MAF more than 0.05 and less than 0.06, with values of beta being positive. These SNPs located in or near genes ligand dependent nuclear receptor corepressor like, non-SMC condensin I complex subunit G (*NCAPG*), leucine aminopeptidase 3, LIM domain binding 2, quinoid dihydropteridine reductase, prominin 1, and transmembrane anterior posterior transformation 1. We found one locus *rs14491030* that located in the missense of the gene NCAPG and another SNP close to NCAPG.

Table 6 showed that the SNPs on GGA2 association with HW association with HW displayed a MAF of ~0.3, with the values of beta being positive. These SNPs located on the intron of sonic hedgehog (*SHH*) and close to genes 5-Hydroxytryptamine receptor 5A, insulin induced gene 1, and limb development membrane protein. The SNPs *rs316413705* explained the highest CPV (3.109%) for HW.

### Linkage disequilibrium analysis

**Figure 3:**
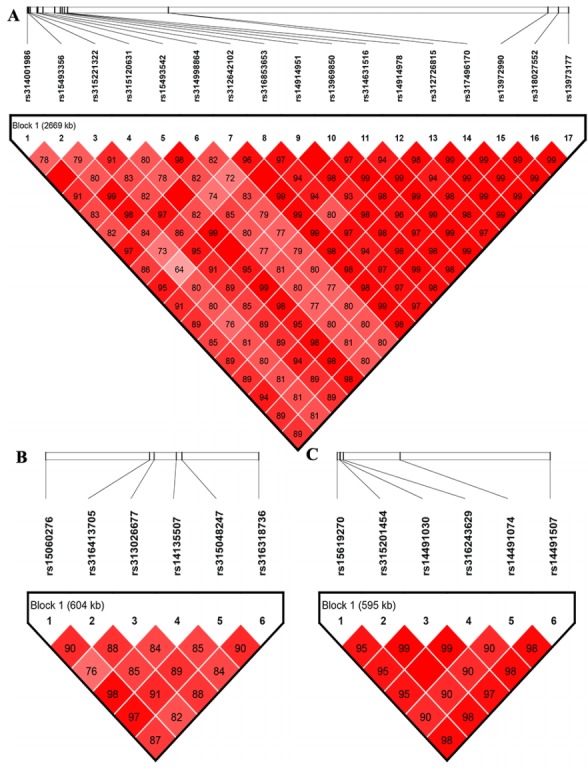
LD plot for significant single nucleotide polymorphisms at GGA1, GGA4, and GGA2. Plot A represents LD analysis on GGA1, plot B represents LD analysis on GGA4, plot C represents LD analysis on GGA2. LD, linkage disequilibrium; GGA, *Gallus gallus* autosome.

The LD analysis was performed because of the potentially strong LD between neighboring variants. The LD analysis results revealed that significant SNPs on GGA1, GGA4, or GGA2 were all extremely strong in a LD status (Figure 3). Combined the aforementioned CPV calculation analysis, LD analysis and gene annotation, we considered *rs312726815*, *rs315120631*, and *rs13972990* on GGA1 as primary candidate loci, with correspond to genes *HTR2A*, *GTF2F2*, and *WDFY2* associated with HW, LW, and PW/GW, respectively. The *NCAPG* gene harbored the missense locus *rs14491030* on GGA4 were considered as a candidate gene for all internal organs, *SHH* gene with intron harbored the SNP *rs14491030* on GGA2 had an association with HW.

### Genome partitioning of genetic variation

**Figure 4:**
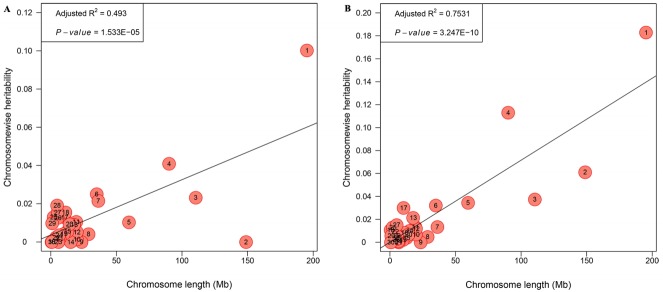
Genome partitioning for LW and GW by joint analysis. The estimated proportion of variance captured by each chromosome against its size. The characters in the circles are the chromosome numbers. Plot A represents liver weight, plot B represents gizzard weight. LW, liver weight; GW, gizzard weight.

The genetic architecture of internal organs was further illustrated by partitioning the genetic variation onto chromosome segments with an exploratory analysis. HW and PW in the joint model could not converge due to the relatively small sample size in the F_2_ population. Only the partitioning spectrum of LW and GW were estimated. The estimates of variance contributed by each chromosome exhibited a medium and strong linear relationship with the length of the chromosome for LW (R^2^ = 0.493, 0.493 Figure 4A) and GW (R^2^ = 0.753, 0.493 Figure 4B). For GGA1 this explained 18.29% and 10.03% of the phenotypic variance for GW and LW, respectively. While for GGA4 it explained 11.30% and 4.09% for GW and LW, respectively.

## DISCUSSION

To accomplish the goal of “500 eggs in 100 weeks”, it is essential to continuous select egg production trait as well as fitness trait (such as increasing heart capacity [18]) for which populations were not routinely selected. Because of the correlation between egg production and fitness showed positive or negative was not absolute, it is essential to detect molecular markers associated with economic traits as well as fitness traits to improve animals simultaneously. Moreover, some body composition trait like gizzard and proventriculus indirectly reflects feed efficiency [5]. Hence, improving these traits will not only make chicken well-being better but also take advantage of these organs. But, these traits are inconvenient or difficult to measure for animals should be killed. Application of DNA markers could simultaneously select economic trait and these traits.

Our results showed that phenotypic data displayed a large variation for all internal weights, which was probably because these traits were not chosen as selection index. The heritability showed a moderate to high, higher than that the previous reports. For LW, the heritability estimates were 0.31 [19] and 0.33 [20] at 6 weeks, and the heritability of HW and GW was 0.27 and 0.44, respectively, in a report by Venturini et al [20]. The difference of the heritability may be due to different breeds and ages [21]. In addition, we dissected genome partitioning of genetic variation for GW and LW. A positive linear correlation between the variance explained by each chromosome and its length were obtained in our work, which corresponds to previous reports [22]. In particular, GGA1 accounted for the largest genetic variance (18.29%) for GW, which is consistent with the highest heritability (0.640), whereas GGA1 accounted for 10.03% genetic variance for LW, the heritability of which is moderate (0.355). We then considered that with the higher heritability of the trait, the genetic variance of GGA will also be higher. This finding is in line with previous research especially in our resource population [23].

Using GWAS method, we totally found 17 SNPs on GGA1 and 6 SNPs on GGA4 associated with all internal organ weights, and 6 SNPs on GGA2 were associated with HW. Previous studies showed that QTL for internal organ weight, such as HW and LW, at 9 weeks was detected on GGA1 and GGA4, respectively [24]. HW and GW at 6 weeks were mapped to GGA13 [10]. The difference between the present study and previous reports might be due to differences in the age and population of the birds used in different studies. These studies used younger birds, the age of the birds in the present study was 72 weeks. In addition, one trait, such as internal organ weight, was controlled by more than one QTL, which will help us understand the genetic architecture underlying the quantitative traits that are controlled by polygenes.

The most significant loci in the GGA1 spanned from 166.99 to 169.66 Mb, which is also related to comb weight [25], egg weight [23], ovary weight [26], and feed intake [27] in our previous studies and growth traits in other reports [28–30]. The internal organs as fitness traits participate in metabolism that affects growth performance and economic traits, therefore the selection of body weight or egg weight will indirectly result in internal organ weight change. This has important ramifications for understanding the pleiotropic effects of the locus or gene [31].

After rigorous statistic and LD analysis, three SNPs *rs3127 26815*, *rs315120631*, and *rs13972990*, correspond to genes *HTR2A*, *GTF2F2*, and *WDFY2*, respectively, were selected as the candidate loci. *HTR2A* was considered as a candidate gene for HW. The mRNA of the gene, which encodes a receptor of 5-hydroxytryptamine (serotonin), is expressed within vascular smooth muscle, endothelial cells, and cardiomyocytes, and plays a role in vasoconstriction and cellular proliferation [32]. Also, the function of the *HTR2A*-encoded protein is associated with blood pressure and heart rate and is a contributing factor to cardiovascular disease in humans [33]. In birds, the size of heart was association with the rate of metabolism and indicates the capacity to move the blood [34]. Therefore, it is reasonable to propose that *HTR2A* was responsible for heart weight, the detailed information needs to be further investigated. Second gene *GTF2F2* nearby SNP *rs315120631* was considered as candidate gene for LW. *GTF2F2* forms a heteromeric general transcription initiation factor [35]. *GTF2F2* widely exists in many tissues and organs, especially in the liver, lung, and kidney. The liver is the main organ regulating body homeostasis [36], where a large amount of active transcription and translation takes place. Reports said that *GTF2F2* is association with rat liver regeneration [37]. Liver is responsible for detoxification and energy balance, which involved in various of biological process including transcription and translation. Hence, further studies about the function of *GTF2F2* in chicken internal organs are needed. The third SNP, *rs13972990*, is near gene *WDFY2*, which is associated with PW and GW. *WDFY2* (WD repeat and FYVE domain containing 2) is an endosomal protein, modulating the *PI3K*/*AKT* pathway, which is known to be involved in oncogenesis [38]. *WDFY2* overexpression can increase adipogenesis, which may play a role in metabolic disorders such as diabetes in humans [39]. Proventriculus and gizzard modulated feed behavior [6] and feed overconsumption [5], indirectly regulating glycogen synthesis. It is tempting to speculate that *WDFY2* may participate in proventriculus and gizzard growth though its involvement is not clear.

Moreover, the region on GGA4 was related to egg weight, eggshell weight, oviduct weight in our population [23,40]. *NCAPG* harbored the missense *rs14491030* was a candidate gene for all internal weight. The gene was related to residual feed intake in cattle [41] and withers height in horses [42] in previous reports. Most breeders focus on egg production in layer chicken, resulting indirectly in selection for fitness trait or digestive organs. *NCAPG* was considered as candidate gene for all internal organ weight though no selection for these traits, pleiotropic effects of the locus on other traits that are under selection, or close linkage and LD with QTL that are under selection [43]. We provide an evidence that quantitative traits that may be controlled by polygenes and a single gene or mutation may lead to a host of alterations in multiple traits.

In addition, GWAS results showed that one QTL on GGA2 associated with HW. The *SHH* could be considered as a candidate gene. The *SHH* gene is one of the Hh proteins, which plays a crucial role in the development of all animals and regulates morphogenesis of a variety of tissues and organs in the embryo [44]. Previous studies showed that *SHH* is necessary for secondary heart field proliferation in humans [45]. Consequently, the function of *SHH* in the chicken heart may involve heart differentiation.

## CONCLUSION

In present study, a GWAS strategy was performed to detect potential QTLs or genes association with internal organ traits. Our study provides evidence that the internal organ weight at 72-weeks old appears to moderate a high heritability and may share the similar genetic mechanisms. Five candidate genes were identified with significant effect on internal weights. These promising loci or genes could be helpful to simultaneous improve layer chicken economic trait and fitness trait to accomplish sustainable use of the chicken.

## Supplementary Data



## Figures and Tables

**Table 1:** Descriptive statistics for internal organ weight in the F_2_ population

Trait	Mean	SD	Min	Max	CV (%)
HW	5.42	1.03	3.00	12.20	19.00
LW	28.18	6.68	12.40	66.80	23.69
PW	1.64	0.79	0.10	14.30	48.36
GW	5.25	1.21	1.00	15.10	22.95

Mean, arithmetic mean; SD, standard deviation; Min, minimum; Max, maximum; CV, coefficient of variance; HW, heart weight; LW, liver weight; PW, proventriculus weight; GW, gizzard weight.

**Table 2:** Summary of genetic analysis for internal organs1)

Trait	HW	LW	PW	GW
HW	**0.283(0.043)**	0.658(0.079)	0.666(0.043)	0.567(0.073)
LW	0.351	**0.355(0.045)**	0.645(0.075)	0.503(0.071)
PW	0.360	0.240	**0.408(0.044)**	0.711(0.049)
GW	0.389	0.358	0.475	**0.640(0.037)**

HW, heart weight; LW, liver weight; PW, proventriculus weight; GW, gizzard weight.

1) Diagonal, heritability estimates; Lower triangle, phenotypic correlations; Upper triangle, genetic correlations. Standard errors of the estimates are in parentheses.

**Table 3:** Genome-wide significant regions identified by genome-wide association study for the weights of internal organ

Trait	GGA	No. snp	Region (Mb)	Trait	GGA	No. snp	Region (Mb)
HW	1	97	165.99–171.60	PW	1	230	165.63–173.78
	2	6	7.79–8.34		4	15	75.48–76.21
	4	42	71.30–77.19				
LW	1	18	167.00–169.72	GW	1	308	165.93–172.12
	4	7	75.48–76.07		4	32	71.30–76.67
Total	1	380	165.63–173.78		4	49	71.30–77.19
Venn diagram	1	17	166.99–169.66		4	6	75.48–76.07

GGA, *Gallus gallus* autosome; SNP, single nucleotide polymorphisms; No. snp, the number of significant SNPs; HW, heart weight; LW, liver weight; PW, proventriculus weight; GW, gizzard weight.

**Table 4:** Information of 17 SNPs on GGA1 and genomic regions to internal organ weight

SNP	Position (bp)	Location (bp)	Candidate/nearest gene	EA/AA	MAF	Beta (SE)				p-value				CPV (%)			
		
						HW	LW	PW	GW	HW	LW	PW	GW	HW	LW	PW	GW
rs314001986	166,994,822	intron/U-3	*GTF2F2*	A/G	0.49	−0.277(0.051)	−0.274(0.054)	−0.309(0.055)	−0.295(0.056)	8.23E-08	4.2E-07	2.41E-08	1.27E-07	3.691	3.819	5.042	4.964
rs15493356	166,998,353	intron	*GTF2F2*	A/T	0.472	−0.256(0.051)	−0.287(0.053)	−0.315(0.054)	−0.278(0.054)	5.49E-07	6.2E-08	6.11E-09	2.84E-07	3.171	4.151	5.264	4.417
rs315221322	167,002,252	intron	*GTF2F2*	G/A	0.479	−0.291(0.051)	−0.284(0.054)	−0.334(0.055)	−0.314(0.055)	1.9E-08	1.4E-07	1.29E-09	1.51E-08	4.04	4.066	5.786	5.496
rs315120631	167,009,025	intron	*GTF2F2*	A/G	0.479	−0.278(0.052)	−0.289(0.054)	−0.335(0.055)	−0.331(0.056)	8.52E-08	9.38E-08	1.54E-09	3.11E-09	3.725	4.219	5.835	6.065
rs15493542	167,042,503	intron	*GTF2F2*	G/A	0.436	−0.267(0.049)	−0.255(0.05)	−0.31(0.051)	−0.292(0.049)	5.08E-08	3.98E-07	1.12E-09	3.32E-09	3.581	3.421	5.147	4.918
rs314998864	167,046,824	intron	*GTF2F2*	G/A	0.471	−0.26(0.051)	−0.284(0.053)	−0.32(0.054)	−0.273(0.054)	4.06E-07	1.02E-07	3.97E-09	5.81E-07	3.269	4.064	5.424	4.3
rs312642102	167,071,397	U_32764	*SPERT*	T/C	0.478	−0.285(0.052)	−0.287(0.054)	−0.323(0.055)	−0.339(0.055)	3.98E-08	1.14E-07	5.33E-09	1.24E-09	3.889	4.149	5.47	6.318
rs316853653	167,129,709	U_5263	*SPERT*	G/C	0.365	−0.287(0.049)	−0.265(0.051)	−0.356(0.051)	−0.377(0.049)	6.01E-09	1.99E-07	5.06E-12	4.33E-14	3.883	3.512	6.376	7.458
rs14914951	167,157,210	D_1359	*SPERT*	T/C	0.491	−0.291(0.052)	−0.281(0.054)	−0.33(0.055)	−0.345(0.056)	1.98E-08	2.38E-07	2.58E-09	7.65E-10	4.044	3.992	5.661	6.538
rs13969850	167,166,474	D_11512	*SPERT*	C/T	0.474	−0.268(0.051)	−0.285(0.054)	−0.336(0.055)	−0.331(0.055)	2.18E-07	1.21E-07	1.1E-09	2.12E-09	3.481	4.128	5.919	6.139
rs314631516	167,176,627	D_27951	*SPERT*	G/A	0.442	−0.307(0.049)	−0.278(0.052)	−0.363(0.052)	−0.366(0.052)	5.6E-10	7.97E-08	5.66E-12	1.76E-12	4.507	4.006	6.813	7.356
rs14914978	167,193,066	U_16438	*HTR2A*	A/T	0.427	−0.303(0.048)	−0.271(0.051)	−0.336(0.051)	−0.33(0.05)	4.74E-10	1.09E-07	8.28E-11	5.7E-11	4.403	3.772	5.856	6.027
rs312726815	167,689,410	U_16831	*HTR2A*	A/C	0.402	−0.314(0.048)	−0.277(0.05)	−0.383(0.05)	−0.399(0.049)	9.46E-11	4.56E-08	4.38E-14	5.29E-16	4.723	3.86	7.363	8.479
rs317496170	167,689,803	D_44012	*WDFY2*	T/A	0.401	−0.313(0.048)	−0.266(0.05)	−0.386(0.05)	−0.403(0.049)	1.13E-10	1.51E-07	2.64E-14	2.47E-16	4.677	3.58	7.454	8.618
rs13972990	169,562,091	D_95255	*WDFY21*	T/C	0.456	−0.314(0.048)	−0.276(0.051)	−0.386(0.051)	−0.44(0.05)	1.27E-10	7.3E-08	5.49E-14	6.9E-18	4.69	3.75	7.312	10.112
rs318027552	169,613,334	D_1533/D_37584	*ALG11/CKAP2*	G/A	0.34	−0.276(0.047)	−0.244(0.049)	−0.351(0.049)	−0.38(0.047)	4.82E-09	6.86E-07	8.44E-13	1.48E-15	3.341	2.781	5.869	7.356
rs13973177	169,663,934	intron/U-3	*GTF2F2/KCTD4*	G/C	0.451	−0.278(0.049)	−0.26(0.051)	−0.358(0.051)	−0.399(0.051)	1.59E-08	4.75E-07	4.63E-12	6.07E-15	3.694	3.351	6.536	8.581

SNP, single nucleotide polymorphism; GGA, *Gallus gallus* autosome; EA, effect allele (minor allele); AA, alternative allele (major allele); MAF, minor allele frequency; Beta, estimated allelic substitution effect per copy of the effect allele; SE, standard error of the beta; CPV, contribution to phenotypic variance (%); HW, heart weight; LW, liver weight; PW, proventriculus weight; GW, gizzard weight. *GTF2F2*, general transcription factor IIF polypeptide 2; *SPERT*, spermatid associated; *HTR2A*, 5-hydroxytryptamine receptor 2A; *WDFY2*, WD repeat and FYVE domain containing 2; *ALG11*, asparagine-linked glycosylation 11; *CKAP2*, cytoskeleton associated protein 2; *GTF2F2*, general transcription factor IIF polypeptide 2; *KCTD4*, potassium channel tetramerization domain containing 4.

**Table 5:** Information of 6 SNPs on GGA4 and genomic regions to internal organ weight

SNP	Position (bp)	Location (bp)	Candidate/nearest gene	EA/AA	MAF	Beta (SE)				p-value				CPV (%)			
		
						HW	LW	PW	GW	HW	LW	PW	GW	HW	LW	PW	GW
rs15619270	75,478,306	D_26163/D_1994	*LCORL/NCAPG*	G/C	0.058	0.571(0.084)	0.437(0.085)	0.466(0.085)	0.531(0.078)	1.58E-11	3.38E-07	5.5E-08	1.52E-11	3.503	1.98	2.369	3.29
rs315201454	75,485,620	intron/D_33477	*NCAPG/LCORL*	A/G	0.058	0.581(0.084)	0.457(0.086)	0.465(0.086)	0.535(0.079)	7.75E-12	1.03E-07	6.7E-08	1.52E-11	3.679	2.189	2.422	3.365
rs14491030	75,486,534	missense/D_34391	*NCAPG/LCORL*	A/G	0.059	0.549(0.083)	0.427(0.084)	0.441(0.085)	0.527(0.077)	6.32E-11	4.86E-07	2.08E-07	1.4E-11	3.284	1.892	2.179	3.28
rs316243629	75,495,451	intron	*NCAPG*	G/A	0.058	0.581(0.084)	0.457(0.086)	0.465(0.086)	0.535(0.079)	7.75E-12	1.03E-07	6.7E-08	1.52E-11	3.679	2.189	2.422	3.365
rs14491074	75,653,956	U_102910	*LDB2*	T/C	0.057	0.558(0.086)	0.479(0.087)	0.493(0.087)	0.574(0.081)	1.16E-10	4.05E-08	1.79E-08	1.66E-12	3.333	2.414	2.636	3.821
rs14491507	76,073,771	U_18141	*PROM1/TAPT1*	G/A	0.051	0.623(0.09)	0.468(0.092)	0.53(0.092)	0.61(0.085)	7.99E-12	3.94E-07	1.06E-08	1.31E-12	3.787	2.075	2.794	3.981

SNP, single nucleotide polymorphism; GGA, *Gallus gallus* autosome; EA, effect allele (minor allele); AA, alternative allele (major allele); MAF, minor allele frequency; Beta, estimated allelic substitution effect per copy of the effect allele; SE, standard error of the beta; CPV, contribution to phenotypic variance (%); HW, heart weight; LW, liver weight; PW, proventriculus weight; GW, gizzard weight. *LCORL*, ligand dependent nuclear receptor corepressor like; *NCAPG*, non-SMC condensin I complex subunit G; *LDB2*, LIM domain binding 2; *PROM1*, prominin 1; *TAPT1*, transmembrane anterior posterior transformation 1.

**Table 6:** Information of 6 SNPs on GGA2 and genomic regions to heart weight

SNP	Position (bp)	Location (bp)	Candidate/nearest gene	EA/AA	MAF	Beta (SE)	p-value	CPV (%)
rs15060276	7,788,887	D_74,602	*HTR5A*	T/C	0.400	0.235(0.045)	2.50E-07	2.489
rs316413705	8,083,684	intron	*SHH*	T/C	0.281	0.282(0.049)	1.35E-08	3.109
rs313026677	8,096,208	U_6,355	*SHH*	A/T	0.383	0.235(0.047)	8.08E-07	2.293
rs14135507	8,159,096	U_69,243	*SHH*	G/A	0.275	0.246(0.048)	4.89E-07	3.027
rs315048247	8,175,293	U_85,440	*SHH*	C/A	0.300	0.271(0.047)	1.49E-08	2.213
rs316318736	8,393,250	U_303,397	*SHH*	A/G	0.302	0.229(0.046)	7.57E-07	2.423

SNP, single nucleotide polymorphism; GGA, *Gallus gallus* autosome; EA, effect allele (minor allele); AA, alternative allele (major allele); MAF, minor allele frequency; Beta, estimated allelic substitution effect per copy of the effect allele (EA); SE, standard error of the beta, which means the effect size of minor alleles; CPV, contribution to phenotypic variance (%); *HTR5A*, 5-Hydroxytryptamine receptor 5A; *SHH*, sonic hedgehog.
