# Supplementary material for: Genetic architecture and candidate genes detected for chicken internal organ weight with a 600 K single nucleotide polymorphism array
Source: Asian-Australas J Anim Sci. 2018 Jul 26;32(3):341–9. doi: 10.5713/ajas.18.0274 (PMC6409475; doi:10.5713/ajas.18.0274)

Supplementary figure

Title: Genetic architecture and candidate genes detected for chicken internal organ weight with a 600 K single nucleotide polymorphism array

Author information

Taocun *Dou* [1,a], Manman *Shen*[1,2,a], Meng *Ma*[1], Liang *Qu*[1,3], Yongfeng *Li*[1], Yuping *Hu*[1], Jian *Lu*[1], Jun *Guo*[1], Xingguo *Wang*[1], and Kehua *Wang*[1,*]

[*]Corresponding author: Kehua *Wang*

Tel: + 86-38-0527-6606, Fax: + 86-0514-8559-9012, E-mail: sqbreeding@126.com

[1] Jiangsu Institute of Poultry Science, Chinese Academy of Agricultural Sciences, Yangzhou, Jiangsu 225216, China

[2] College of Animal Science and Technology, Yangzhou University, Yangzhou, Jiangsu 225009, China

[3] College of Animal Science and Technology, Nanjing Agriculture University, Nanjing, Jiangsu 210095, China

[a] Taocun Dou and Manman Shen have contributed equally to this study.

ORCID

Kehua *Wang* https://orcid.org/0000-0003-4125-1204

Supplementary Figure S1. Manhattan plot and QQ-plot of genome-wide association analysis for LW (liver weight). The left plot is the Manhattan plot, which shows the –log10 (observed p values) for association of single nucleotide polymorphisms (y-axis) plotted against their chromosomal positions on each chromosome (x-axis). The right plot is the QQ test for population structure, the x-axis indicates the expected –log10-transformed p values, and the y-axis shows the observed –log10-transformed p values.

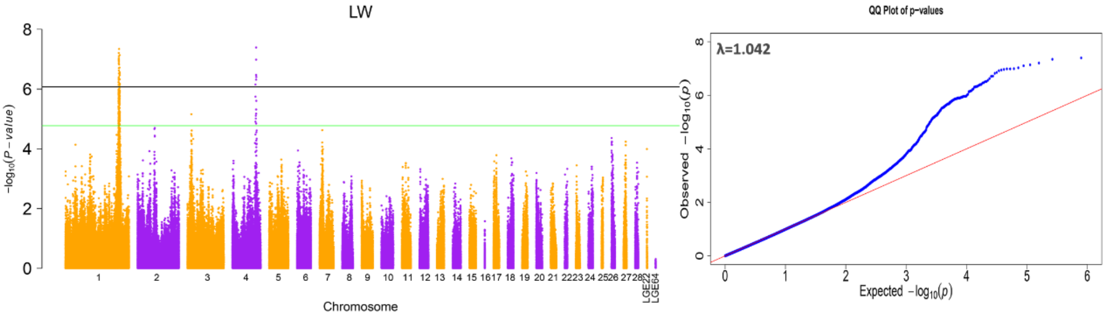

Supplementary Figure S2. Manhattan plot and QQ-plot of genome-wide association analysis for PW (proventriculus weight). The left plot is the Manhattan plot, which shows the –log10 (observed p values) for association of single nucleotide polymorphisms (y-axis) plotted against their chromosomal positions on each chromosome (x-axis). The right plot is the QQ test for population structure, the x-axis indicates the expected –log10-transformed p values, and the y-axis shows the observed –log10-transformed p values.

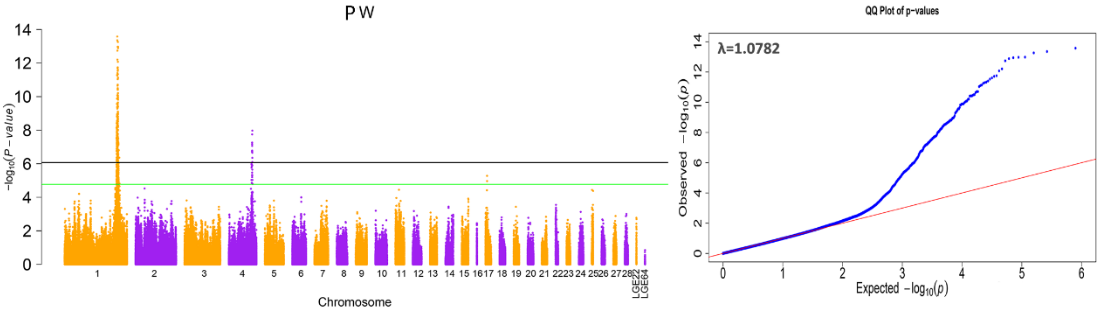

Supplementary Figure S3. Manhattan plot and QQ-plot of genome-wide association analysis for GW (gizzard weight). The left plot is the Manhattan plot, which shows the –log10 (observed p values) for association of single nucleotide polymorphisms (y-axis) plotted against their chromosomal positions on each chromosome (x-axis). The right plot is the QQ test for population structure, the x-axis indicates the expected –log10-transformed p values, and the y-axis shows the observed –log10-transformed p values.

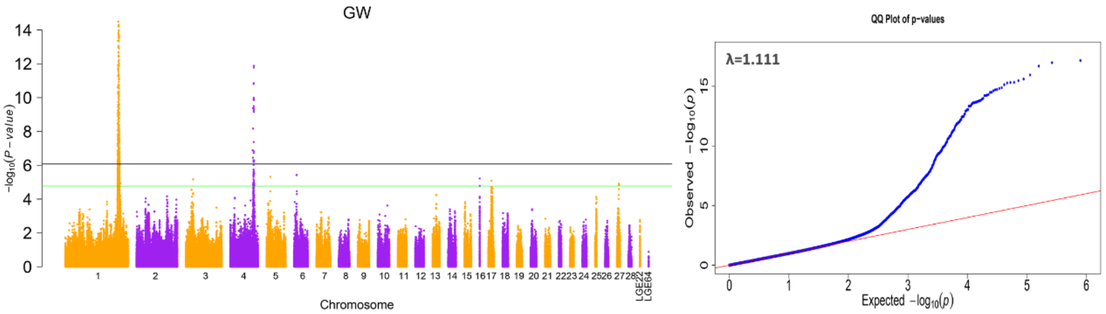

Supplement: Supplementary file 1 [file ajas-18-0274-supplementary.pdf]
